# Book Review: Good Morning, I Love You: Mindfulness and Self-Compassion Practices to Rewire Your Brain for Calm, Clarity, and Joy

**DOI:** 10.3389/fpsyg.2021.691673

**Published:** 2021-05-07

**Authors:** Chandan Kumar Srivastava, Rashmi Gupta

**Affiliations:** Cognitive and Behavioural Neuroscience Laboratory, Department of Humanities and Social Sciences, Indian Institute of Technology Bombay, Mumbai, India

**Keywords:** mindfulness, self-compassion, intention, attitude, attention

It is undeniable that mindfulness practices do bring wonders; however, surprisingly, many fail to continue it in the long run. The dropouts seem to constantly judge themselves for not being perfect in practice and compare themselves with someone better at it. We wondered what was missing; we stumbled upon Dr. Shauna Shapiro's book, “Good Morning, I love you,” which scientifically addresses the missing gap in practice. Dr. Shapiro is a professor, author, and internationally acclaimed expert in mindfulness and compassion. The author's decades of research, insights, and own personal practices led her to discover what stops people from experiencing mindfulness's benefits. She suggested that mindfulness's full potential is difficult to realize unless practiced with the right attitudes such as compassion and curiosity.

In Chapter 1, the author kick-starts with her personal story by discussing how through practicing mindfulness and self-compassion, she regained her faith to start living her life once again, having undergone major surgery in her spine due to scoliosis, which had almost shattered her dreams and hopes to be fully able to live her life as it was before. She suggested that mindfulness is not just about being in the current moment, but it's about welcoming our present experience with an attitude of kindness, curiosity, and compassion.

In chapter 2, the author puts on the scientific lens on mindfulness to harness the power of neuroplasticity (the brain's ability to form neural connections adaptively over time). Neuroplasticity helps in installing novel/useful habits/behavior we want to cultivate and eliminate those we are wrestling to give up.

In chapter 3, the author busted the top 10 myths (e.g., mindfulness is only to reduce stress, it takes too much time, it makes you soft, it is only for Buddhist, etc.) by carefully presenting scientific evidence. She insisted on incorporating mindfulness in our day-to-day life rather than only in challenging situations (e.g., stressful situations). She further elaborated that mindfulness enhances the awareness of moment to moment experience making it enlivening and enriching. This view is in line with research findings, suggesting that mindfulness improves attention processing (Norries et al., 2018) that effectively analyses a situation.

In chapter 4, the author introduced her first scientific model of mindfulness, which suggests that intention, attention, and attitude are the right ingredients of fulfillment, transformation, and joy (Shapiro et al., 2006). The *intention* component helps us resist the temptation of being reactive by reminding us what is of utmost importance in a given moment. The *attention* component allows us to be focused on a task at hand whilst avoiding distractions/multitasking. The *attitude* (kindness, curiosity, and compassion) component helps us cultivate a positive attitude toward ourselves and others. In other words, she highlighted the significance of positive emotion processing in our life. This view is in line with scientific findings, suggesting that the processing of positive emotions enhances well-being (Fredrickson, 2004).

In chapter 5 and 6, the author suggested mindfulness helps us cultivate self-compassion that teaches us to be warm and understand ourselves when we suffer. Self-compassion enhances cognitive control and resiliency, helping to deal with challenging situations effectively (Neff and Germer, 2018). She further insisted on deploying our attention toward positive information and using adaptive (e.g., self-compassion) rather than maladaptive (e.g., shame, self-blame) coping mechanisms to regulate negative emotion. This view is in line with recent research, which suggested that negative information processing consumes all attention resources (Gupta, 2019, for a review) that shut down the brain's learning centers.

In chapter 7, the author further extended other deeply rooted adaptive mechanisms (e.g., acceptance, shifting perspective, appraisal, etc.) of mindfulness to regulate our emotions. She also highlighted the global effect of mindfulness: “forgiveness,” a conscious, deliberate decision to release feelings of resentment toward ourselves or others. Forgiveness removes the emotional barrier to relate with ourselves and others by freeing us from deeply buried emotions, heartbreaks, and burdens we carry in our hearts.

An evolutionary perspective suggests that our brain gives more weightage to negative information compared to positive information. However, in chapter 8, the author further revisited the significance of positive emotions in our life. The “happiness set-point theory” suggests that we cannot change our happiness baseline (Brickman and Campbell, 1971). She challenges this theory by offering a bundle of mindfulness practices to raise the bar of happiness. More specifically, she suggested the inclusion of genuine smile, gratitude, generosity, empathy, joy, kindness, seeing the good in others, etc., in our day to day life. In fact, in chapter 9, the author further insisted on enriching the life experience through mindfulness in daily life, such as eating, parenting, working, decision-making, etc.

In the last couple of chapters, the author highlighted that mindfulness makes us more connected with each other and the world around us, which breaks the illusion of loneliness. Finally, she encourages readers to embrace the “Good morning; I love you” practice rooted in mindfulness that strengthens the neural pathway of self-love by cultivating compassion for oneself and others.

In a nutshell, this book is a practical manual trying to weave neuroscience research and mindfulness to rewire our brain for happiness, compassion, and well-being. Pursuing mindfulness with the right attitude is the author's formula for persistent mindfulness practice to access its benefits. This book emphasizes that mindfulness is much more than just paying attention in the present moment; instead, it is about paying attention with an attitude of kindness, curiosity, and compassion. While mindfulness has been exceptional in getting rid of negative emotion, the role of mindfulness in processing positive emotion is left unexplored. The current need is not only to study how mindfulness brings about positive emotion, but also how it affects the processing of positive emotion itself. For example, it would be interesting to see if mindfulness can eliminate addictive behavior by filtering irrelevant rewarding information. Such a balanced approach studying mindfulness in the context of both negative and positive emotion would help in understanding the complete picture of emotion processing in mindfulness.

## Author Contributions

All authors listed have made a substantial, direct and intellectual contribution to the work, and approved it for publication.

## Conflict of Interest

The authors declare that the research was conducted in the absence of any commercial or financial relationships that could be construed as a potential conflict of interest.
